# An Eclectic Reviewer

**Published:** 1893-03

**Authors:** 


*Atlanta Medical and Surgical Journal:*

### AN *ECLECTIC* REVIEWER.

We cannot let the opportunity slip to make our grateful acknowledgments to the Massachusetts *Medical Journal*, an Eclectic publication which emanates from over-cultivated Boston, for the quiet compliment in its January issue. Our own December JOURNAL contained a review of Dr. McClellan's late *Regional Anatomy*, and the January number of our Eclectic contemporary from New England "reviewed" the same work. The reviews furnished a beautiful instance of coincidence of ideas and showed how easily two minds may run in the same channel, especally when they travel *tandem*. For instance:

| ATLANTA MEDICAL AND SURGICAL JOURNAL, December, 1892. | Massachusetts *Medical Journal*, January, 1893. |
| --- | --- |
| The second volume of Dr. McClellan's work fully sustains the high excellence of the first. The regions of the abdomen, perineum, back, pelvis, groin, hip, thigh, knee and popliteal space, leg, ankle and foot are herein described in that same happy manner which characterized the text of the first volume. All through this work we believe the reader will be struck with this feature—the simple and pleasing style in which the author describes what is ordinarily regarded as a dry subject. . . . . . . . . | The second volume of Dr. McClellan's work fully sustains the high excellence of the first. The regions of the abdomen, perineum, back, pelvis, groin, hip, thigh, knee and popliteal space, leg, ankle and foot are herein described in that same happy manner which characterized the text of the first volume. All through this work the reader will be struck with the simple and pleasing style in which the author describes what is ordinarily regarded as a dry subject. . . . . . . . . . . . . . |
| There is some nomenclature which differs from that of our standard text book (Gray), and which may be commended. The external and internal abdominal rings are called, respectively, the superficial and deep openings. The tensor *vaginæ* femoris is called (by preference) the tensor *fasciæ*. This name conveys to the beginner a better idea of the function of the muscle, besides creating no suggestion of any relation with another part which may not be very distant, etc., etc. | We notice some names not found in Gray, the use of which we heartily commend. The external and internal abdominal rings are called, respectively, the superficial and deep openings. The tensor *vaginæ* femoris is called (by preference) the tensor *fasciæ*. This name conveys to the beginner a better idea of the function of the muscle, besides creating no suggestion of any relation with another part, which may not be very distant, etc., etc. |

Indeed it would seem that plagiarism had struck bottom when one medical journal descends to appropriate a *book review* from another. To review properly a work of the magnitude and excellence of the one above referred to requires time and patience, and it is often a not very agreeable occupation. In the present instance we were more than usually careful, because of the nature of the work, and at the publisher's request. Naturally, therefore, we very much object to having the results of our labor, even in so small a matter as this, stolen by some one who probably had neither the brains nor application for the duty of reviewer. Our *un*-esteemed huckleberry contemporary from New England should be ashamed!

